# Supplementary material for: Validity of Prediction Equations of Maximal Heart Rate in Physically Active Female Adolescents and the Role of Maturation
Source: Medicina (Kaunas). 2019 Nov 13;55(11):735. doi: 10.3390/medicina55110735 (PMC6915545; doi:10.3390/medicina55110735)
Supplement: Supplementary file 1 [file medicina-55-00735-s001.pdf]

# Supplementary material

**Table S1.** Comparison (paired-samples *t*-test) of squared differences (predicted-actual $HR_{max}$) by maturation level.

| Variable | Total ($n$ = 71) | Less Matured ($n$ = 37) | More Matured ($n$ = 34) |
|---|---|---|---|
| Fox - Actual HRmax ($bpm^2$) | 119.6 ± 172.9 * | 107.1 ± 171.7 [a] | 133.2 ± 175.7 [b] |
| Tanaka - Actual $HR_{max}$ ($bpm^2$) | 80.2 ± 110.6 | 81.5 ± 89.6 | 78.7 ± 131.1 |

*difference between the two variables at $p = 0.043$. [a] $p = 0.340$ (comparison between the two variables). [b] $p = 0.060$ (comparison between the two variables).

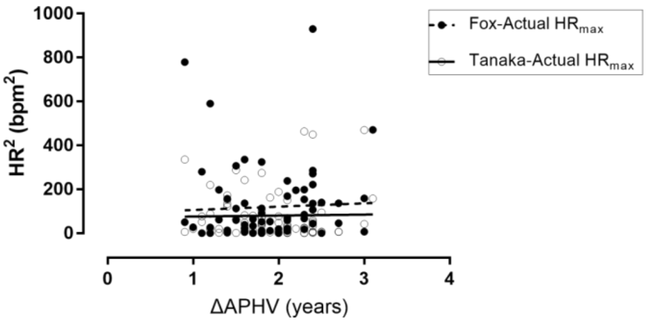


Fox-Actual $HR_{max}$: Y = 15.14 × X + 90.81 R = 0.04, $p = 0.7116$

Tanaka-Actual $HR_{max}$: Y = 4.435 × X + 71.77 R = 0.02, $p = 0.8657$

**Figure S1.** Single scatter plot with maturation on the horizontal x-axis, and the (squared) differences (Delta HR) on the y-axis.

## Mixed models analysis

### Syntax

mixed $HR_{max}$ with Assessment_method by Maturation_group
/fixed Assessment_method Maturation_group Assessment_method * Maturation_group
/print = solution
/method = mL
/random intercept | subject(id)

**Table S2.** Estimates of Fixed Effects [a].

| Parameter | Estimate | Std. Error | df | t | Significance | 95% Confidence Interval Lower Bound | 95% Confidence Interval Upper Bound |
|---|---|---|---|---|---|---|---|
| **Intercept** | **202.29** | **162** | **213** | **124.506** | <0.001 | 199.09 | 205.49 |
| Assessment_method | −0.84 | 0.75 | 213 | −1.122 | 0.263 | −2.33 | 0.64 |
| [Maturation_group = 1.00] | 3.03 | 2.25 | 213 | 1.346 | 0.180 | −1.41 | 7.47 |
| [Maturation_group = 2.00] | 0 [b] | 0 | . | . | . | . | . |
| [Maturation_group = 1.00] * Assessment_method | −0.85 | 1.04 | 213 | −0.812 | 0.418 | −2.90 | 1.21 |
| [Maturation_group = 2.00] * Assessment_method | 0 [b] | 0 | . | . | . | . | . |

[a] Dependent Variable: HRmax. [b] This parameter is set to zero because it is redundant.
